# Cardiac Complications and COVID-19: A Review of Life-threatening Co-morbidities

**DOI:** 10.2174/011573403X279782240206091322

**Published:** 2024-02-20

**Authors:** Zeinab Eftekhar, Habib Haybar, Alireza Mohebbi, Najmaldin Saki

**Affiliations:** 1 Thalassemia & Hemoglobinopathy Research Center, Health Research Institute, Ahvaz Jundishapur University of Medical Sciences, Ahvaz, Iran;; 2 Atherosclerosis Research Center, Ahvaz Jundishapur University of Medical Sciences, Ahvaz, Iran;; 3 Department of Hematology and Blood Banking, School of Allied Medical Sciences, Tehran University of Medical Sciences, Tehran, Iran

**Keywords:** COVID-19, SARS-CoV-2, heart failure, myocardial infarction, arrhythmia, cardiac complication

## Abstract

The novel 2019 coronavirus disease (COVID-19) was first reported in the last days of December 2019 in Wuhan, China. The presence of certain co-morbidities, including cardiovascular diseases (CVDs), are the basis for worse outcomes in patients with COVID-19.

Relevant English-language literature was searched and retrieved from the Google Scholar search engine and PubMed database up to 2023 using COVID-19, SARS-CoV-2, Heart failure, Myocardial infarction, and Arrhythmia and Cardiac complication as keywords.

Increased hemodynamic load, ischemia-related dysfunction, ventricular remodeling, excessive neurohumoral stimulation, abnormal myocyte calcium cycling, and excessive or insufficient extracellular matrix proliferation are associated with heart failure (HF) in COVID-19 patients. Inflammatory reaction due to the excessive release of inflammatory cytokines, leads to myocardial infarction (MI) in these patients. The virus can induce heart arrhythmia through cardiac complications, hypoxia, decreased heart hemodynamics, and remarkable inflammatory markers. Moreover, studies have linked cardiac complications in COVID-19 with poor outcomes, extended hospitalization time, and increased mortality rate. Patients with COVID-19 and CVDs are at higher mortality risk and they should be given high priority when receiving the treatment and intensive care during hospitalization.

## 1 INTRODUCTION

The novel 2019 coronavirus disease (COVID-19), which was caused by the severe acute respiratory syndrome coronavirus-2 (SARS-CoV-2), was first reported in the last days of December 2019 in Wuhan City, Hubei Province, China [1-3]. It was spreading rapidly and became a global emergency due to its high mortality rate [4]. As of 1 November 2022, about 630,452,398 cases have been confirmed around the world, and about 6,590,213 deaths have been attributed to the COVID-19 pandemic crisis. The presence of certain co-morbidities, including cardiovascular diseases (CVDs), is the basis for worse outcomes in patients with COVID-19. For this reason, the presence of such underlying diseases in these patients can potentially weaken the prognosis of patients [5]. Patients with pre-existing CVD appear to be more vulnerable to COVID-19 and may experience a higher mortality rate [1, 2, 4, 6-8]. Given the growing concern over CVD comorbidity in COVID-19 patients and the lack of a clear understanding of the mechanisms underlying these complications in COVID-19 patients, we made the decision to examine the research and theories presented in this context. Additionally, we summarized the results of observational studies that have been published between 2020 and the time this review was written (Table **1**).

## 2 MATERIALS AND METHODS

Relevant English-language literature was searched and retrieved from the Google Scholar search engine and PubMed database up to 2023 using COVID-19, SARS-CoV-2; Heart failure, Myocardial infarction, Arrhythmia and Cardiac complication as keywords.

### 2.1 Heart Failure

Heart failure (HF) is a clinical syndrome caused by structural and functional defects in the myocardium, which results in impaired ventricular filling and reduced ability of the heart to pump blood [9, 10]. Increased hemodynamic load, ischemia-related dysfunction, ventricular remodeling, excessive neurohumoral stimulation, abnormal myocyte calcium cycling, and excessive or insufficient extracellular matrix proliferation are some of the main pathogenic mechanisms leading to HF [9, 11]. HF patients are at special risk for severe infections due to reduced immunity, general weakness, and hemodynamic ability [8], about a quarter of hospitalized COVID-19 patients had a new onset of HF; it also has been observed in about one-third of those admitted to the intensive care unit (ICU) despite not having a history of HF [8, 12]. Various mechanisms, including myocardial ischemia or infarction, increased oxygen demand, elevations in pulmonary pressure, pulmonary embolism, myocarditis, stress cardiomyopathy, and cytokine storm, cause COVID-19 patients to need enhanced heart function and output. However, if the patient has an HF background, his cardiovascular system will not be able to compensate for this complicated situation. According to studies conducted in HF patients, monocytes have been shown to produce more Tumor necrosis factor (TNF)-α and less Interleukin (IL)-10 than healthy subjects, which, in combination with the widespread systemic inflammatory response associated with severe COVID-19 infection, worsening the patient condition [8, 13-15]. Angiotensin-converting enzyme 2 (ACE2) is a membrane-bound aminopeptide that has a specific expression on the surface of lung alveolar epithelial cells and, to a lesser extent, in the heart. ACE2 is also expressed on the surface of venous and arterial endothelial cells, arterial smooth muscle, kidney tubular epithelium, and small intestine epithelium [16, 17]. Based on investigations, the spike (S) glycoprotein receptor binding domain (RBD), which is one of the surface glycoproteins of SARS-CoV-2, can bind to ACE2 and facilitate the virus’s entry to its target cell [12, 14, 16, 18]. The RBD subunit of the S protein causes the virus to bind to the target cell, and the S2 subunit causes the fusion of the virus with the membrane of the target cell [12]. The specific binding between RBD and ACE2 creates an opportunity for developing novel therapeutic products, such as antibodies or small-molecule inhibitors, for targeting this interaction in COVID-19 patients [16]. ACE inhibitors (ACEI), angiotensin II receptor blockers (ARBs), and angiotensin receptor-neprilysin inhibitors are the most important guideline-based medical treatments for patients with chronic systolic HF [14]. However, there is a dispute over the actual effectiveness of these therapeutic inhibitors in COVID-19. Some researchers believe that there is no significant association between the use of ACEI or ARBs, and increased risk of contracting COVID-19 or disease severity [14, 18], while others, based on analysis of COVID-19 patients, stated that the risk of mortality has been decreased in COVID-19 patients who used ACEI and ARBs as a treatment for their hypertension [19]. Another group of researchers believes in a theory that these inhibitors with up-regulation of ACE2 facilitate the virus’s entry into the cells at a larger number, and cardiovascular patients must be off these drugs. However, currently, there is no proof supporting this theory [20]. Due to the positive effect of these drugs in managing hypertension and the lack of convincing evidence for their role in an increased risk of infection and mortality in COVID-19 patients, it seems reasonable for patients with hypertension to continue using them.

### 2.2 Myocardial Infarction

Myocardial Infarction in the pathology of myocardial infarction (MI), it is defined as myocardial cell death, which is necrosis of the coagulation band and/or contraction due to prolonged ischemia, which usually evolves through oncosis, but it can be caused to a lesser degree by apoptosis, too [21, 22]. MI is associated with an inflammatory reaction due to higher expression of IL-6 and IL-1β and TNF-α that is triggered by mechanical deformation, ischemic stimulus, reactive oxygen species (ROS), and cytokine self-amplification pathways [23, 24], which in the acute post-infarction period they lead to the survival or death of myocytes, modulation of cardiac contractility, remodeling of the vascular endothelium, and employment of additional circulating inflammatory cells to the damaged myocardium so we have more local oxidative stress and regeneration, but also the initiation of wound healing processes which is a precondition for scar formation [24]. In the chronic postinfarction period, the longtime presence of cytokines activates matrix metalloproteinases and collagen formation and accelerates regeneration processes [24, 25]. It should be considered that the increased secretion of cytokines may worsen the SARS-CoV-2 infection and/or even lead to secondary infections [26]. Cardiac troponin I (cTnI) and T (cTnT) are exclusive components of the contractile apparatus of myocardial cells for the heart [27, 28]. Measurement exceeding the 99^th^ percentile upper limit of a normal reference population shows an elevated cTn value essential to diagnosing acute MI [22, 27-30]. It has been demonstrated that an increase in troponin levels is associated with poor prognosis in COVID-19 patients with non-ST-segment elevation acute MI [31]. Acute myocardial infarction (AMI) is divided into two categories: ST-elevated MI (STEMI) and non-ST-segment elevation acute MI (NSTEMI) [32]. Based on studies, the case fatality rate among patients with both COVID-19 and STEMI was reported to be substantially higher (28.6%) compared with all other STEMI patients (11.9%) [33]. In comparison between patients within or out-of-hospital STEMI, a concomitant diagnosis of COVID-19 was remarkably associated with higher rates of in-hospital mortality compared with patients without a diagnosis of COVID-19 from the past year [34]. Further research is needed to figure out the potential mechanisms of this association.

### 2.3 Arrhythmia

Arrhythmias are defined as abnormal rates or rhythms of the heartbeats. There are several kinds of arrhythmia with various appearances, exceedingly slow or fast heartbeats such as sinus bradycardia and atrial tachycardia, irregular rhythm with missing or distorted wave segments and intervals, or both [35]. Altered intercellular coupling, interstitial edema, and cardiac fibrosis lead to abnormal conduction in addition to abnormal Ca^2+^ control and reduced K+ channels, leading to repolarization abnormalities and action potential conduction abnormalities [36, 37]. Gaaloul *et al*. reported that myocardial inflammation caused by viral infection leads to ion exchange dysfunction or electrophysiological and structural remodeling as a mechanism for arrhythmia [37, 38]. Arrhythmia is not an early or common manifestation of COVID-19, and most symptoms are related to respiratory system involvement [37, 39]. However, among the arrhythmias, atrial arrhythmias are the most common kind of arrhythmia seen in COVID-19 patients [40]. It has been said that 12% of arrhythmias in COVID-19 patients might be related to atrioventricular (AV) blockage [40, 41]. Taking together, it seems that the occurrence of arrhythmia in COVID-19 patients is due to multiple factors, including hypoxia due to acute respiratory distress (ARD), cardiac complication, decreased heart hemodynamics, remarkable inflammatory response, direct invasion of the virus and using QT-prolonging drugs [40, 42-44]. Hydroxychloroquine and azithromycin, which were briefly used for the treatment of COVID-19 patients, can induce QT prolongation and, subsequently, cardiac death and ventricular cardiac arrhythmia. Therefore, these drugs are no longer being used for patients [40, 45]. One of the direct mechanisms of these drugs for QT prolongation is the blocking of fast delayed rectifier potassium currents (IKr or hERG) [40]. While sinus tachycardia has been reported to be secondary to a physiological response to viral infection or drug side effects, sinus bradycardia is commonly seen in patients with COVID-19. It even takes up to 2 weeks [37, 39, 46, 47]. Acute bradycardia in COVID-19, which could be the result of direct infection of cardiomyocytes and cardiac conduction system by SARS-CoV-2, is associated with elevated inflammatory markers [48]. IL-6 is one of such inflammatory markers that plays a major role in cytokine storm observed in COVID-19 patients and causes the elevation in vagal tone, decreased variability in heart rate, and eventual bradycardia by directly affecting the sinoatrial node [49]. Therefore, evaluating the levels of this pro-inflammatory cytokine could have prognostic values in these patients.

### 2.4 Atrial and Ventricular Fibrosis

Atrial fibrosis (AF) is the most common clinical cardiac arrhythmia [50]. This condition could be the result of the TGF-β1 signaling that makes atrial fibroblast/myofibroblast cells produce collagen. On the other hand, the natriuretic peptide receptor clearance (NPR-C) in response to this signaling would increase the sensitivity of atrial cells and elevate the risk of AF. Ventricular fibroblasts have fewer NPR-C and, therefore, are more resistant to fibrosis [51]. Based on recent studies, it has been observed that there is a significant difference between the levels of TGF-β1 in COVID-19 patients and controls [51]. The increased levels of TGF-β1 in COVID-19 patients could be due to excessive production by lung tissue, neutrophils, or activated macrophages during infection by SARS-CoV-2. Accordingly, measuring the levels of TGFβ1 could serve as a prognostic marker and predict the severity of disease in COVID-19 patients [51]. Also, a significant incidence of new-onset AF has been reported in COVID-19 patients [52]. Possible mechanisms for the onset of AF include cytokine storm, decreased protection of the cardiovascular system due to reduction in ACE2 activity by the virus, loss of fluid-electrolyte homeostasis, atrial structural change due to interaction between CD147 and sialic acid-spike protein, and finally reactive oxygen species which could cause oxidative damage to the atrial. Subsequently, it seems that these mechanisms can induce myocardial injury and remodeling [53].

### 2.5 Ischemic & Non-ischemic Cardiomyopathy

Various stages of myocardial dysfunction and fibrosis were discovered in ischemic and non-ischemic cardiomyopathies [54]. Ischemic cardiomyopathy (ICM) is defined as myocardial dysfunction caused by the spread of severe coronary artery stenosis or chronic coronary artery occlusion that commonly leads to heart enlargement, arrhythmia, and HF with disruption in cardiomyocyte metabolism, coronary artery stenosis, coronary embolism, vasospasm, vascular endothelial dysfunction, and microcirculatory disorders [55]. Recent studies showed an uprising in the incidence of stress cardiomyopathy during the COVID-19 pandemic compared to the pre-COVID-19 era [56]. Extended investigation on stress cardiomyopathy cases in COVID-19 patients with a history of hypertension or diabetes (who had proper treatment and did not require hospitalization before COVID-19 infection) demonstrated that despite the lack of history of CVD in these cases, all of them showed chest pain, aside from common viral infection findings, for 10 days before hospital admission [57]. It has been hypothesized that SARS-CoV-2 with a direct toxic effect on myocytes, enhances the ability of its surface protein to bind ACE2 and therefore facilitate its entry to these cells [58]. Aggregation of multiple factors, including hypersensitivity of cardiomyocytes to sympathetic stimulations with COVID-19, would eventually result in a kind of cardiomyopathy called Takotsubo syndrome, which is associated with left ventricular dysfunction [57, 58].

### 2.6 Thrombotic Events

Due to the presence of various factors, including endothelial dysfunction, inflammation, Oxidative stress, and platelet activation, the probability of occurrence of thrombosis in arterial and venous circulation in COVID-19 patients is higher [19, 59]. High rates of coagulation abnormalities and thrombotic events have been seen through intensive care studies with COVID-19 patients from multiple European centers [9, 60, 61]. ACE2 converts angiotensin II to angiotensin (1-7), resulting in a decreased level of angiotensin II and an elevated level of angiotensin (1-7) [62]. Since angiotensin (1-7) has anti-inflammatory and anti-thrombotic activities while showing antioxidant properties *via* inducing the production/releasing of nitric oxide from endothelial cells, therefore, ACE2 plays a key role in regulating these functions by converting angiotensin II to angiotensin (1-7) [62-64]. Thus, with decreased activities of ACE2 due to its interaction with SARS-CoV-2, we can expect an incidence of inflammation and increased risk of clot formation in COVID-19 patients.

On the other hand, due to decreased antioxidant levels and subsequently increased reactive oxygen activities, we can observe damage to endothelial cells, followed by overexpression of P-selectin, loss of the ability of the endothelium to maintain coagulation cascade, and eventually increased risk of clot formation [62, 65]. Thrombocytopenia in patients with COVID-19 can be due to multiple factors, including the direct effect of the virus on bone marrow and abnormal hematopoiesis, inhibiting platelet synthesis, destruction of platelets by the immune system, and increased platelet aggregation and consumption [66, 67]. In the direct invasion of the virus on bone marrow cells, the virus probably uses CD13 and CD66a, which are expressed on the surface of CD34+ hematopoietic stem cells, megakaryocytes and its precursors, and platelets, as receptors and enters these cells, inhibiting their growth and causing apoptosis [68]. As mentioned earlier, due to the high production of inflammatory cytokines, a cytokine storm occurs in COVID-19 patients, which destroys tissues, including the lung tissue and its endothelium, which is associated with the activation, aggregation, and consumption of platelets. Finally, it can lead to thrombocytopenia [68]. In addition, some of these inflammatory cytokines, such as TGF-β, IFN-α, and platelet factor 4 (PF4), can also inhibit the production of megakaryocytes [68]. Also, considering that liver cells play an important role in the synthesis and secretion of thrombopoietin (TPO) and ACE2 is also expressed on the surface of liver cells, it is possible that the SARS-CoV-2 virus destroys hepatic cells through interaction with ACE2 and decrease the production and secretion of TPO, thereby causing thrombocytopenia [68]. In addition, thrombocytopenia can also occur through drugs, such that antiviral drugs, ribavirin, and fluoroquinolones, which are used to treat COVID-19, can directly through the inhibition of hematopoiesis and indirectly the destruction of TPO-producing liver cells, cause thrombocytopenia in patients [68]. Platelet count can be useful as a prognostic marker for COVID-19, as a decrease in platelet count (thrombocytopenia) increases the risk of severe COVID-19 and death up to 3 times [67]. According to the studies, D-dimer level is significantly associated with arterial thrombotic complications, which include MI, acute and subacute limb ischemia that is not prevented by thromboprophylaxis, and stroke [69]. Also, treatment with low molecular weight heparin (LMWH) has been associated with lower mortality in people with high D-dimer levels [69, 70]. On the other hand, a correlation between the increased level of D-dimer and the mortality rate in patients with COVID-19 has been seen [69, 71]. Since the previously mentioned unusual arterial thrombotic complications have been observed in patients with COVID-19, the measurement of D-dimer serum level can be more appropriate to use as a predictive marker of arterial thrombotic complications and identify people at risk [69, 72]. It also seems that three coagulation parameters, Prothrombin Time (PT), Activated Partial Thromboplastin Time (aPTT), and International Normalized Ratio (INR), along with red cell distribution width (RDW) index in COVID-19 patients are among the strong indicators in predicting mortality caused by COVID-19 in children because in a study these parameters showed high power for mortality prediction in hospitalized children with COVID-19 [73]. According to a recent study, it has been found that arterial thrombotic complications occur in those patients with COVID-19 who had severe inflammation or the location of the thrombosis was unusual in them [72].

### 2.7 COVID-19 Vaccination Association with CVDs

Based on the reports, the vast majority of patients who developed cardiovascular symptoms after receiving the COVID-19 vaccine were men [86]. It has also been said that myocarditis and pericarditis are mainly seen in mRNA vaccines such as Pfizer, Moderna, and MI, and heart attacks are more common with the AstraZeneca vaccine [87]. It is possible that myocarditis after the injection of the vaccine, which mainly occurs after 72 hours of the injection of the second dose and more in young people, occurs due to the similarity of the vaccine with the proteins of the heart cells, which leads to a cytokine response and an autoimmune reaction in the patient [88]. There are various hypotheses regarding the association between AMI and the COVID-19 vaccines, as some believe that receiving the COVID-19 vaccine can cause a tendency towards ischemia ending in a cardiovascular event. AMI is observed mainly in older adults about 24 hours after the first dose of the vaccine. The onset of AMI can be due to the tendency of elderly people to be poly-morbid, stress caused by vaccine injection, an autoimmune reaction against platelets, or attributed to allergic vasospasm after injection or Kounis syndrome because it seems that a high amount of IgE antibody can be one of the risk factors for AMI occurrence [86, 88, 89]. A few cases of vaccine-associated immune thrombosis and thrombocytopenia (VITT) have been reported in people vaccinated with the AstraZeneca vaccine, which is believed to be caused by the interaction of the vaccine with platelets or PF4, followed by platelet activation [90]. Laboratory diagnosis methods for this complication include the use of enzyme-linked immunosorbent assay (ELISA) to check for the presence of antibodies against PF4, as well as heparin-induced platelet activation assay (HIPA) or serotonin-release assay (SRA) to check the function of platelets [90, 91]. To disrupt prothrombotic processes and prevent thrombosis in people with VITT, high-dose intravenous immunoglobulin (IVIG) or dexamethasone can be used [90].

## 3 COMORBIDITIES AND CARDIAC INJURIES

### 3.1 Pre-existing CVDs

As mentioned before, SARS-CoV-2 can bind to ACE2 on the surface of heart and lung cells, and by preventing the function of ACE2, it prevents the control of the renin-angiotensin-aldosterone system, or in other words, it prevents the protective function of ACE2 for the cardiovascular system. It also can cause respiratory symptoms by infiltrating the alveolar epithelial cells [41, 92]. On the other hand, it causes the release of pro-inflammatory cytokines [93, 94]. Altogether, these could lead to heart disorders and dysfunction [93]. According to studies, people with a history of CVD are at greater risk and worse progression of COVID-19 than other people [4, 7, 95]. The case fatality rate (CFR) in patients with COVID-19 investigated in the cohort study who did not have a history of underlying disease was 2.3%, and in patients with COVID-19 with a history of CVD, it was increased to 10.5% [7]. This increased sensitivity of cardiovascular patients to COVID-19 can be related to the increased release of ACE2 in these patients compared to healthy individuals [92].

## 4 DIABETES MELLITUS

Compared to healthy controls, patients with a history of diabetes are at a higher risk of contracting COVID-19 with a significant increase in COVID-19-associated mortality. Based on studies conducted in China and Italy, the prevalence of diabetes in patients who died due to COVID-19 is about twice that of patients who have recovered from this disease [95-98]. In another study, researchers monitored several COVID-19 patients and reported that the mortality rate in the general population which was 2.3%, while this rate was 7.3% in diabetic patients [2, 99]. As mentioned earlier, infection with SARS-CoV-2 causes the release of pro-inflammatory cytokines such as IL-6 in diabetic patients, leading to a more intense cytokine storm in them [96, 100]. Factors such as the virus’s higher binding affinity. As a result, an easier entry into the cell, the reduction of virus clearance and decreased T cells function may also increase the sensitivity of COVID-19 in diabetic patients [5]. Since viral infections can cause significant changes in blood glucose levels and thus hurt disease control, perhaps this is why the association of SARS-CoV-2 with diabetes causes a worse prognosis of COVID-19 in diabetics [100]. It is believed that the increased glucose levels are due to direct infection of pancreatic beta cells by SARS-CoV-2 and decreased insulin secretion or insulin resistance in diabetic people [5, 101]. Another way that COVID-19 can cause an increase in the blood sugar level in diabetic patients is the presence of hypoxia and cell lysis during the infection, which leads to an increase in the lactate levels and lactate can increase the sugar level by affecting the glucogenesis cycle [5]. Based on the findings, the level of fibrin and D-dimer in diabetic patients is higher than in healthy people, leading to hyper-coagulopathy in these patients and can increase the severity of COVID-19 in them [100, 102]. Since the increased level of LDH has a direct relationship with the severity of the disease, LDH can be used as a prognostic biomarker with 100% sensitivity in patients with COVID-19 [5]. Management of patients, rapid diagnosis, triage, and quarantine of patients should be undertaken, and patients should be monitored according to the severity of the disease. Some of them, such as patients with COVID-19 who have high troponin levels, may need ICU or cardiac care. An important point is to avoid unnecessary diagnostic tests [7]. One way to manage COVID-19 is to strengthen the immune system as well as manage the use of the suppressors of the immune system and inflammatory responses in the body, in which healthy nutrition plays a major role [103, 104]. Patients with low oxygen pressure can benefit from various supportive measures. These measures include respiratory support and oxygen therapy. Additionally, certain medications can be used to alleviate symptoms. For example, diuretics can help reduce edema, anticoagulants can prevent thromboembolism, and drugs that increase cardiac output can be beneficial [3, 9, 105-107]. Xanthenone, which is an ACE2 activator, reduces blood pressure and strengthens heart function by preventing kidney and heart fibrosis in spontaneous hypertension [107]. Since the renin-angiotensin system plays an important role in the occurrence of acute respiratory distress syndrome (ARDS), which can also be seen in COVID-19 patients, it is plausible to consider the potential therapeutic use of this medication in the future for treating COVID-19 patients [108]. American College of Cardiology (ACC) recommends that COVID-19 patients with a history of coronary artery disease (CAD) should use statins, ACE2 inhibitors, beta-blockers, and aspirin to protect the cardiovascular system [107] Regarding the control of diabetic patients with COVID-19, it should be noted that most people with type 2 diabetes have high blood pressure and lipids, so it is important to use antihypertensive and lipid-lowering regimens to control the disease [109]. The use of metformin and sodium/glucose cotransporter 2 (SGLT-2) inhibitors in diabetic patients with COVID-19 should be discontinued due to the risk of diabetic ketoacidosis and hyperglycemic diabetic ketoacidosis. Still, it is not recommended to stop their use in people with diabetes who have no signs of infection and only for prophylaxis. [109].

## 5 DISCUSSION

As the COVID-19 pandemic spread around the world in late 2019 and early 2020, the medical community and healthcare systems faced an unprecedented challenge. At the same time as establishing relative stability in hospitals and centers involved with patients, researchers had the opportunity to investigate the pathogenesis of the disease and its relationship with common co-morbidities such as malignancies, metabolic disorders (*e.g.*, diabetes mellitus), and CVDs. They reported that there is an association between the infection of COVID-19 in patients with these co-morbidities and a poorer prognosis. Among the cardiac injuries seen in COVID-19 patients, HF is due to the reduction of hemodynamic capacity, immunodeficiency, and general weakness. As a result, a higher risk of infection has been observed more closely [8]. Among the mechanisms of HF incidence in COVID-19 patients, we can mention increased hemodynamic load, ischemia-related dysfunction, ventricular remodeling, excessive neurohumoral stimulation, abnormal myocyte calcium cycling, and excessive or insufficient extracellular matrix proliferation [9, 11]. COVID-19 patients are also at risk of developing MI, because, basically, this cardiac complication is associated with an inflammatory reaction caused by increased levels of inflammatory cytokines, which, ironically, is also observed in the pathogenesis of COVID-19 [23, 24]. Viral infections can cause arrhythmia with mechanisms, such as ion exchange dysfunction or electrophysiological and structural remodeling [37, 38]. Infection with SARS-CoV-2 can also cause arrhythmia through hypoxia, reduction of cardiac hemodynamics, cardiac complication, significant inflammatory response, and direct invasion of the virus [40, 42-44]. Atrial fibrillation, as a common arrhythmia in COVID-19, can occur due to various conditions such as increased cytokine levels, decreased cardiovascular protection from ACE2-virus interactions, changes in electrolytes and the structure of the atrium caused by the interaction of CD147 and sialic acid-spike protein [53]. During the COVID-19 pandemic, we also observed an increase in stress cardiomyopathy [56]. Due to endothelial dysfunction, extensive inflammation, oxidative stress, and platelet activation, the occurrence of thrombotic events in arteries and veins in COVID-19 is expected. This problem reminds the necessity of monitoring coagulation parameters in the laboratory, especially since some of them effectively predict mortality in patients [73]. Studies have shown that in some cases, some types of COVID-19 vaccines have been associated with heart problems, including ischemia and AMI. However, more research is needed to determine the precise mechanism underlying these events; in the interim, vaccination appears to be the most effective way to end the COVID-19 pandemic. It is necessary to keep in mind that the presence of a history of cardiovascular complications and metabolic disorders such as diabetes mellitus in a COVID-19 patient increases the risk and worsens the prognosis compared to people without a history. It can help formulate treatment strategies and approach to the patients [7, 95, 96, 110].

## CONCLUSION

According to our investigation, based on several studies that have been done in this field, there is a significant association between cardiac complications in COVID-19 and poor outcomes, increased hospitalization time, and mortality rate in patients. But due to insufficient understanding of different aspects of this relationship, it seems that in the future, more studies are needed to determine and identify the underlying mechanisms involved in this association.

## Figures and Tables

**Table 1:** An overview of the results of observational studies published between 2020 and the time this review was written.

**Cardiovascular Disease (CVD)**	**No. of Participants**	**M/F Ratio (%)**	**Age**	**Findings**	**references**
Heart failure (HF) & ischemic heart disease	Total: 859 With HD: 113	50.5/49.5 55.8/44.2	68.1 75.6	Heart Disease (HD) was not independently related to prognosis. Statins were associated with decreased mortality independently (*p* = 0.023). Cardiovascular events during hospitalization were associated with poor outcomes (*p* = 0.007) Ischemic heart disease was the most common type of HD (63%) HF was the most common event during hospitalization (7.4%)	[74]
	Total: 283 Before COVID-19 (BC): 164 After COVID-19 (AC): 119	NR 51/49 59/41	NR 82 80	There was a considerable but statistically non-significant drop in referrals before the first UK death due to COVID-19 compared with those referred after (AC) (*p* = 0.06). Only age (hazard ratio 1.04, *p* = 0.03) and AC cohort status (hazard ratio 2.1, *p* = 0.017) remained significant predictors of mortality.	[75]
QTc prolongation in hospitalized COVID-19 patients	Total: 279 QTc prolongation: 69	52/48 57/43	62 67	Patients with QTc prolongation were older (*p*=0.003) with more chance of having underlying CVD (*p*= 0.001) Patients who were on angiotensin-converting enzyme inhibitors (ACEi) were less likely to develop QTc prolongation (*p*= 0.014). QTc prolongation was not associated with increased ventricular arrhythmias or mortality. QTc prolongation was associated with higher odds of having a new bundle branch block (OR 5.09, 95% CI 2.14–12.08), arrhythmia during hospitalization (OR 3.00, 95% CI 1.49–6.02), elevated troponin level (OR 2.08, 95% CI 1.11–3.90) and type 2 myocardial infarction (MI) (OR 2.46, 95% CI 1.07–5.66). Hydroxychloroquine was associated with higher odds of developing QTc prolongation (OR 2.10, 95% CI 1.20–3.80)	[76]
Acute PVT in cirrhotic patients with COVID-19	Total: 70 With COVID-19: 28	NR 60.7/39.3	NR 46	Cirrhotic patients with COVID-19 infection showed a higher incidence of PVT compared to patients without the infection (*p*<0.05) Fatigue was the most common clinical presentation in COVID-19, COVID-19-infected group (82.1%) followed by fever (78.5%)	[77]
Thromboembolic and bleeding events in COVID-19 patients in ICU	Total: 1369 With thrombosis: 158 With bleeding: 309	68/32 77/23 72/28	68 66 68	Thromboembolic events were not associated with mortality (*p* = 0.14), but bleeding events were (*p* = 0.002). CRP and D-dimer levels were associated with thrombosis in ICU patients (*p*<0.01) Ischemic heart disease and HF were found in 11% and 3% of patients with thrombosis, respectively.	[78]
Thromboembolic events (TEE) in outpatients with COVID-19	Patients with TEE: 141471	43.2/56.8	46.1	The most common comorbidities were hypertension, hyperlipidemia, anxiety, diabetes, and depression. Cognitive HF, ischemic stroke, MI, DVT, and PE were found in 15.9%, 12.5%, 8.9%, 10.4%, and 8.8% of patients, respectively. The incidence of thromboembolic events was 1.4% and <1% of patients died.	[79]
Thrombotic events in anticoagulant users with COVID-19	Total: 20360 Exposed to oral anticoagulant: 10180	48/52 NR	79.9 NR	Patients who were exposed to oral anticoagulant (OAC) showed a lower risk of death (IRR 0.60, 95% CI 0.55–0.65) OAC patients had a higher risk of hospital admission (IRR 1.16, 95% CI 1.03–1.29) OAC patients had a higher risk of stroke and pulmonary embolism (IRR 1,80, 95% CI 1.06–3.06).	[80]
CV complications included acute cardiac injury in, HF, cardiogenic shock, and acute coronary syndrome	Total: 108	65/35	51	Patients with acute cardiac injury had higher mortality than those without (16/28 [57.1%] *vs.* 14/78 [17.5%]; *p* < 0.0001). Multivariate logistic regression analysis showed that acute cardiac injury (OR: 11.3), lymphopenia (OR: 4.91), use of inotropic agents (OR: 2.46), and neutrophil/lymphocyte ratio (OR:1.1) were independent predictors of mortality.	[81]
Cardiac involvement in patients recovered from COVID-19	Total: 26 Positive CMR: 15 Negative CMR: 11	38/62 27/73 55/45	38 39 37	Native T1, T2, and ECV were all found to be significantly elevated in patients with positive conventional CMR findings, compared with patients without positive findings and controls (*p* = 0.002; *p* < 0.001, and *p* = 0.002, respectively)	[82]
acute MI (AMI)	27 427 emergency department visits or hospitalizations were analysed	NR	NR	In-hospital mortality increased for AMI during COVID-19 OR, 1.46; 95% CI, 1.21–1.76),	[83]
ST-segment elevation myocardial infarction (STEMI)	Total: 1788 COVID-19 era: 733 Pre-COVID-19: 1055	NR 75/25 77/23	NR 62 61	COVID-19-positive patients had higher mortality (28% *vs*. 6%, *p* < 0.001) and cardiogenic shock (20% *vs*. 7%, *p* < 0.001) in comparison to COVID-19-negative ones.	[84]
90-day post-discharge mortality in patients hospitalized for MI and COVID-19	Control: 55060 Group A: 135 Group B: 329	73/27 71/29 68/32	67 71 69	In-hospital and 90-day post-discharge mortality rates of patients with previous COVID-19 were higher than patients without concomitant/previous confirmed COVID-19 (OR adjusted in-hospital 1.83, 95% CI: 0.97—3.46; OR adjusted post-discharge 0.77, 95% CI 0.28—2.13). Patients with concomitant COVID-19 showed excess cardiac complications during hospitalization (OR adjusted 1.62, 95% CI 1.29—2.04), in-hospital mortality (OR adjusted 3.31, 95% CI 2.32—4.72), and 90-day post-discharge mortality (OR adjusted 2.09, 95% CI 1.24—3.51).	[85]

**Abbreviations:** HF: Heart failure; HD: Heart disease; NR: Not reported; OR: Odds ratio; CI: confidence interval; PVT: portal vein thrombosis; CMR: cardiac magnetic resonance; CV: cardiovascular; incidence rate ratio (IRR).

## LIST OF ABBREVIATIONS

COVID-19: 2019 Coronavirus Disease
SARS-CoV-2: Severe Acute Respiratory Syndrome Coronavirus-2
CVDs: Cardiovascular Diseases
HF: Heart Failure
ACE2: Angiotensin-Converting Enzyme 2
RBD: Glycoprotein Receptor Binding Domain
ACEI: ACE-inhibitors
ROS: Reactive Oxygen Species
cTnI: Cardiac Troponin I
cTnT: Cardiac Troponin T
STEMI: ST-Elevated MI
NSTEMI: Non-ST-Segment Elevation Acute MI
AV: Atrioventricular
AF: Atrial Fibrosis
NPR-C: The Natriuretic Peptide Receptor Clearance
ICM: Ischemic Cardiomyopathy
LMWH: Low Molecular Weight Heparin
aPTT: Activated Partial Thromboplastin Time
INR: International Normalized Ratio
AMI: Acute Myocardial Infarction
VITT: Vaccine-Associated Immune Thrombosis and Thrombocytopenia
ELISA: Enzyme-linked Immunosorbent Assay
HIPA: Heparin-Induced Platelet Activation Assay
SRA: Serotonin-Release Assay
ARDS: Acute Respiratory Distress Syndrome
ACC: American College of Cardiology
CAD: Coronary Artery Disease
SGLT-2: Sodium/Glucose Cotransporter 2
